# Network analysis of frontal lobe alpha asymmetry confirms the neurophysiological basis of four subtypes of depressive behavior

**DOI:** 10.3389/fpsyt.2023.1194318

**Published:** 2023-06-28

**Authors:** Christopher F. Sharpley, Vicki Bitsika, Wayne M. Arnold, Shabah M. Shadli, Emmanuel Jesulola, Linda L. Agnew

**Affiliations:** Brain-Behavior Research Group, University of New England, Armidale, NSW, Australia

**Keywords:** depression, behavior, subtypes, frontal lobe asymmetry, network analysis

## Abstract

**Introduction:**

Although depression is widespread carries a major disease burden, current treatments remain non-universally effective, arguably due to the heterogeneity of depression, and leading to the consideration of depressive “subtypes” or “depressive behavior subtypes.” One such model of depressive behavior (DB) subtypes was investigated for its associations with frontal lobe asymmetry (FLA), using a different data analytic procedure than in previous research in this field.

**Methods:**

100 community volunteers (54 males, 46 females) aged between 18 yr. and 75 years (*M* = 32.53 yr., SD = 14.13 yr) completed the Zung Self-rating Depression Scale (SDS) and underwent 15 min of eyes closed EEG resting data collection across 10 frontal lobe sites. DB subtypes were defined on the basis of previous research using the SDS, and alpha-wave (8-13 Hz) data produced an index of FLA. Data were examined via network analysis.

**Results:**

Several network analyses were conducted, producing two models of the association between DB subtypes and FLA, confirming unique neurophysiological profiles for each of the four DB subtypes.

**Discussion:**

As well as providing a firm basis for using these DB subtypes in clinical settings, these findings provide a reasonable explanation for the inconsistency in previous FLA-depression research.

## Introduction

The common definition of Major Depressive Disorder (MDD) is based on nine heterogeneous major symptoms (1). When combined with the Associated Features of MDD, there are nearly 1,500 clusters of these symptoms that may qualify a person for a diagnosis of MDD (2), leading to the argument for using diagnostic models that include particular clusters of symptoms that are called depression “subtypes.” It is important to note that these are not always legitimate forms of MDD because they may not include the required list of Diagnostic Criteria for MDD, which is at least (i) either Depressed mood or Anhedonia, plus (ii) sufficient of the remaining seven Diagnostic Criteria to make a total of five symptoms (1). Instead, “depressive subtypes” is used as a term to define groups of MDD Diagnostic Criteria and Associated Features that may be defined according to some characteristic that is common to all the depressive symptoms in the cluster, but does not necessarily meet the full diagnosis of MDD (3, 4). Although “depression subtypes” has been used in this way in the previous literature (3–5), it is more accurate to refer to these clusters as “depressive behavior” subtypes (DB subtypes), and that term will be used here. Regardless of the term used, these models of depression are investigated because they may hold greater promise for valid identification and treatment of this heterogeneous disorder than a simple unitary diagnosis of “depression” (6–8).

Several models of DB subtypes have been developed, often based upon the neurophysiological underpinnings of clusters of MDD symptoms (4, 9, 10). One such set of DB subtypes that has been proposed on the basis of neurophysiological substrates as well as the clinical coherence of symptoms is: depressed mood; anhedonia; cognitive depression; and somatic depression (11, 12). Table 1 describes the allocation of items derived from a standardized self-report scale (the Zung Self-rated Depression Scale: 13) to each of these four DB subtypes, and shows that each of those subtypes was distinct in terms of the symptoms attributed to it. That allocation of different MDD symptoms provides a *prima facie* case for their orthogonality, and some previous data have verified that in differing populations (13–16).

**Table 1:** Four depression subtypes and relevant Zung SDS^1^ items [from Sharpley and Bitsika (11)].

Subtype	Depressed mood	Anhedonia	Cognitive depression	Somatic depression
SDS items	1. I feel downhearted and blue 3. I have crying spells or feel like it 14. I feel hopeful about the future 15. I am more irritable than usual 17. I feel that I am useful and needed 19. I feel that others would be better off if I were dead	5. I eat as much as I used to 6. I still enjoy sex 18. My life is pretty full 20. I still enjoy doing the things I used to	11. My mind is as clear as it used to be 12. I find it easy to do the things I used to do 16. I find it easy to make decisions	4. I have trouble sleeping at night 7. I notice that I am losing weight 8. I have trouble with constipation 9. My heart beats faster than usual 10. I get tired for no reason 13. I am restless and cannot keep still

1 Zung Self-rated Depression Scale.

However, although content and hypothesized neurophysiological substrates of DB subtype symptoms provide an initial basis for considering those subtypes as distinct from each other and from MDD in general, confirmation of those assumptions via neurophysiological data could provide a further validation of them, and provide support for their use in clinical settings. One of the most-studied neurological substrates for MDD is “Frontal Lobe Asymmetry” (FLA), representing differences in the level of electrical activity between the left and right frontal lobes of the human brain (17–21). Specifically, this hypothesis argues that depressed individuals will demonstrate greater levels of electrical activity in the right frontal lobe than in the left frontal lobe than non-depressed individuals (19, 22–30). The rationale for this right > left (R > L) hemisphere dominance in electrical activity as a correlate of MDD is that the behavioral inhibition system (BIS), which initiates withdrawal from aversive stimuli (31–33), is thought to be most strongly represented by electrical activity in the right frontal hemisphere, whereas the behavioral approach system (BAS), responsible for engaging with pleasant stimuli, is thought to be best represented by electrical activity in the left frontal hemisphere (32–34). Although some other frequencies of electrical activity are occasionally measured in FLA-depression research, the major metric used has been EEG data at the 8–13 Hz frequency (known as “alpha” waves) (22, 23, 34), which are indicative of the mentally relaxed state, distinct from electrical activity at higher frequencies (e.g., beta waves at 13 to 18 Hz) which are associated with more intense mental activity (35). Alpha activity is thus used as an inverse indicator of actual activity in the relative brain sites.

The association between the BIS and MDD is argued as based upon the depressed individual’s desire to withdraw from specific aversive environmental stimuli (via dominance of the BIS over the BAS), representing a behavioral strategy for reducing the total negative emotional experiences encountered (36–41). There are some data which support the association between the BIS and MDD (42–44), although the hypothesis has been challenged (45). Further, a meta-analysis of 26 studies concluded that the association between FLA and the BAS was “considerably weaker and more inconsistent than generally assumed” [(46), p. 167]. That is, while some data support the FLA-MDD hypothesis [e.g., (47–49)], other data are non-supportive [e.g., (50)]. Thus, the hypothesized association between FLA and DB subtypes represents a potentially valuable avenue of research to inform clinical practice, because it follows the recommendation to investigate the association between FLA and “symptom clusters rather than diagnoses” [(51), p. 26].

Methods of detecting significant and meaningful associations between FLA and depression have traditionally relied upon correlational and regression procedures, which can provide an indication of the amount of variance in a particular DB subtype that is accounted for by the FLA across specific EEG sites (52). While these are valid procedures, a recent statistical model that describes those associations using a different process avoids some of the limitations of purely correlational methods, and may provide a different perspective on how FLA is associated with DB subtypes. That model is “network analysis,” which describes the causal interplay between variables rather than simply the correlation coefficients between them (53). Some previous studies using network analysis of MDD symptoms have provided valuable insights into the ways that the nine symptoms of MDD are related, with implications for focused treatment of those symptoms [e.g., (54, 55)]. Adding EEG data to this kind of analysis could clarify the association between FLA and DB subtypes, and thus contribute to the debate regarding the FLA-MDD link.

Therefore, this study aimed to evaluate the FLA-depression hypothesis by network analysis, using FLA data from 10 frontal and using alpha activity (i.e., 8–13 Hz) as the major indicator of FLA, and four DB subtypes as the indices of depression. Due to the lack of previous network analyses incorporating this kind of FLA and DB subtype data, no directional hypotheses could be set at the specific DB subtype level, apart from the general expectation that there would be a confirmation of the FLA-MDD hypothesis described above.

## Materials and methods

### Participants

100 adult volunteers (54 males, 46 females) aged between 18 and 75 years (*M* age = 32.53 yr., SD = 14.13 yr) were recruited from the New England region of New South Wales, Australia, selected on the basis of no previous medical history of severe physical brain injury, brain surgery, history of epilepsy or seizure disorder, or claustrophobia (EEG data were collected in a small booth). Because 61 to 70% of left-handed people also have left hemispheric dominance (56, 57), and the BAS or BIS are not associated with handedness (58, 59), handedness was not a selection criteria. This study was approved by the Human Research Ethics Committee of the University of New England, Australia (Approval No. HE14-051), and all participants gave written consent. Because the study was focused upon community participants, medication status was not collected in the data.

### Scale

The Zung Self-Rating Depression Scale (SDS) (60) includes 10 positively-worded and 10 negatively-worded questions which have been developed from factor analytic studies of MDD (1). Respondents indicate the frequency of each of the 20 SDS depressive symptoms during the last 2 weeks by answering in one of four possible ways: “None or a little of the time” (score = 1), “Some of the time” (2), “Good part of the time” (3), or “Most or all of the time” (4). Total raw scores range from 20 to 80 (60, 61), and raw scores of 40 or above indicate the presence of “clinically significant depression” [(61), p. 335]. The SDS has split-half reliability of 0.81 (60), 0.79 (62), and 0.94 (63), with an internal consistency (alpha) of 0.88 for depressed patients and 0.93 for non-depressed patients (64). Participants’ mean scores were calculated on each of the four DB subtypes using the method described by Sharpley and Bitsika (11) and shown in Table 1, based upon the diagnostic criteria for MDD (1).

### EEG data

EEG data were collected via a 40-channel Digital EEG Amplifier (NuAmps), using a *Quick Cap* with electrodes, during continuous EEG measurement of 3 min Eyes Closed resting condition. EEG sites were cleaned with *Nuprep* gel, plus an alcohol swab before fitting the cap. All electrode impedances were checked to ensure that they were <5 KΩ. Participants sat in the experimental booth and their EEG data were collected with *Neuroscan* amplifier and a desktop computer. EEG signals were acquired and recorded using the *Curry 7* software. EEG data from only the 10 Frontal lobe electrode data are reported here because of the focus of this study upon FLA. EEG data were collected at a sampling rate of 1 KHz and the frequency band was set to collect alpha wave activity using low and high filters of 8 and 13 Hz, respectively. The allowable impedance level in each electrode was set as <5 KΩ using the 10–10 electrode placement system and the CAR (Common Average Referencing) referencing style.

Data were processed using a low filter (high pass), frequency of 1 Hz and a slope of 2 Hz; a high filter (low pass) with frequency of 30 Hz and a slope of 8 Hz; a notch filter of 50 Hz (Harmonics) with a slope of 1.5 Hz; and a band stop filter of frequency of 50 Hz (Harmonics) with a width of 10 Hz and slope of 5 Hz. Data tapering was done using a Hann window with a 10% width to prevent data loss. Data were visually examined to identify artefacts (eye movements, muscle movements, spontaneous discharges or electrode pops, etc.), which were then removed from the data record. Bad block and eye blink detection (using the magnitude of eye blink deflections as a set threshold criterion to detect artefacts) was undertaken by three automated methods (Subtraction, Covariance and Principal Component Analysis) to produce clean EEG data.

Back-to-back epochs of 4 s duration were then created from the cleaned EEG data (bad blocks were excluded from averaged data). Most participants had over 90% usable artefact-free epochs for both Eyes Opened and Eyes Closed conditions. EEG data were then digitally filtered for alpha-band frequencies (8–13 Hz). Spectral analysis was performed on the generated epochs (for both conditions for each participant) with a Fast Fourier Transformation (FFT) to calculate the power spectra. The power values obtained from FFT were averaged across the 4-s EEG epochs. From this process, the total power within the alpha (8–13 Hz) frequency range was obtained for each condition for each participant. The values of the total power within the alpha (8–13 Hz) frequency range were then extracted and transferred to an SPSS file for statistical analysis. Alpha EEG asymmetry was calculated from the log transformed alpha power values obtained from corresponding cerebral sites, i.e., LogRight α minus LogLeft α (65–68). By subtracting the left hemisphere alpha activity from the right hemisphere alpha activity, a *negative* result indicates greater alpha activity in the left hemisphere, and a *positive* result indicates greater alpha activity in the right hemisphere. Because alpha activity equates to lower levels of actual activation (69, 70), then a positive (alpha-based) result indicates there is less overall activation in the right hemisphere, whereas a negative (alpha-based) result indicates greater overall activation in the right hemisphere. Thus, a negative alpha-based result indicates dominance of the BIS system over the BAS system (hypothesized to correlate with depression); a positive alpha-based result indicates dominance of the BAS over the BIS (hypothesized to correlate with absence of depression).

### Procedure

Participants read an Explanatory Statement and signed a Consent Form, and completed a background questionnaire (age, sex) and the SDS. Participants’ scalps were then prepared and the electrode cap fitted. Headphones were placed on participants so as to minimize the effect of external stimuli. Following 15 min of sitting still (adaptation), the audio-recorded verbal instructions for the experimental protocol (20 min adaptation, 3 min Eyes Closed) was presented via headphones to ensure consistency across participants.

### Statistical analyses

Networks were estimated, using RStudio (71), both with and without regularization, because recent methodological literature suggests unregularized models are better suited to the exploratory detection of edges in restricted sample sizes (72, 73). The comparison of networks with and without regularization has been performed in other depression biomarker network analyses [e.g., (74)]. The regularized Guassian graphical models (GGMs) were estimated using *bootnet* (74), with LASSO regularization using the Extended Bayesian Information Criterion (EBIC) tuning parameter (i.e., gamma level), initially set at 0 to maximize sensitivity to detect small-sized edges (75), and then run again at gamma levels 0.25 and 0.5 as a form of sensitivity analysis to determine which edges were retained in these more conservative models (76). The unregularized GGM model was estimated using ggmModSelect in *bootnet* at a gamma level of 0 with stepwise model selection. Spearman correlation matrices were used in the estimation of all networks. Stability analyses of the network parameters (centrality and edges) were performed with *bootnet* using nonparametric bootstrapping with 2,500 samples. Predictability (i.e., the proportion of variance of each node explained by its neighboring nodes with which it shares a non-zero edge) was calculated using *mgm* (77). Network structure visualizations were drawn with *qgraph* (78). Reporting standards recommended by Burger et al. (79) were followed.

## Results

There was no statistical difference between the ages of the males and females *F*(1,99) = 0.131, *p* = 0.718, *η*^2^ = 0.001, nor their SDS total scores *F* = 0.165, *p* = 0.685, *η*^2^ = 0.002, their four DB subtype scores (all *p* > 0.557), or any of the five sets of FLA data (all *p* > 0.085), allowing male and female data to be analyzed together. There was no significant correlation between age and SDS total score (*r* = 0.006, *p* = 0.954), or age and any of the four DB subtypes (all *p* > 0.178), or any of the FLA data (all *p* > 0.170). The mean SDS score was 36.7 (SD = 11.26, range = 21 to 66), and 33 participants met Zung’s (61) criteria for “clinically significant depression.” The mean SB subtype scores are shown in Table 2, with paired *t*-test results, indicating that all possible paired combinations of the four DB subtypes were significantly different. Additionally, Table 2 presents the Pearson correlations with the total SDS score for each DB subtype (all *p* < 0.001). Internal consistency for the SDS (Cronbach’s alpha) was 0.905, and ranged from 0.678 to 0.861 for the four DB subtypes, but it is sometimes difficult to obtain acceptable Cronbach alpha values for scales with fewer than 10 items, and so the mean inter-item correlations were observed, and fell within the range 0.354 to 0.572, indicative of reasonably robust relationships between items within DB subtypes and allowing further data analysis. Although there was some (non-significant) evidence for skewness towards the lower end of the scale for the SDS total scores, this is to be expected in a community sample, and inspection of the Normal Q-Q plots for the SDS revealed an almost completely straight line, suggestive of normality. Therefore, SDS scores were able to be used untransformed in data analysis. Kolmogorov–Smirnov statistics for all EEG site data were significant (*p* < 0.05), and therefore all raw EEG values underwent log transformation. There were no significant correlations between the SDS total score and any of the EEG FLA scores (all *p* > 0.223).

**Table 2:** Mean scores for four DB subtypes, paired *t*-tests and Pearson correlation coefficients with SDS total score.

DB subtype pair	Mean	SD	*t*	*p*	r*
Depressed mood Anhedonia	1.736 1.850	0.635 0.677	−0.121	0.036	0.921 0.678
Cognitive depression	2.183	0.810	−9.912	<0.001	0.831
Somatic depression	1.618	0.511	2.728	0.008	0.748
Anhedonia Cognitive depression			−6.676	<0.001	
Somatic depression			4.330	<0.001	
Cognitive depression Somatic depression			10.180	<0.001	

* Pearson correlation coefficients for DB subtypes and SDS total score.

### Network analysis of FLA EEG data and DB subtypes

**Figure 1:**
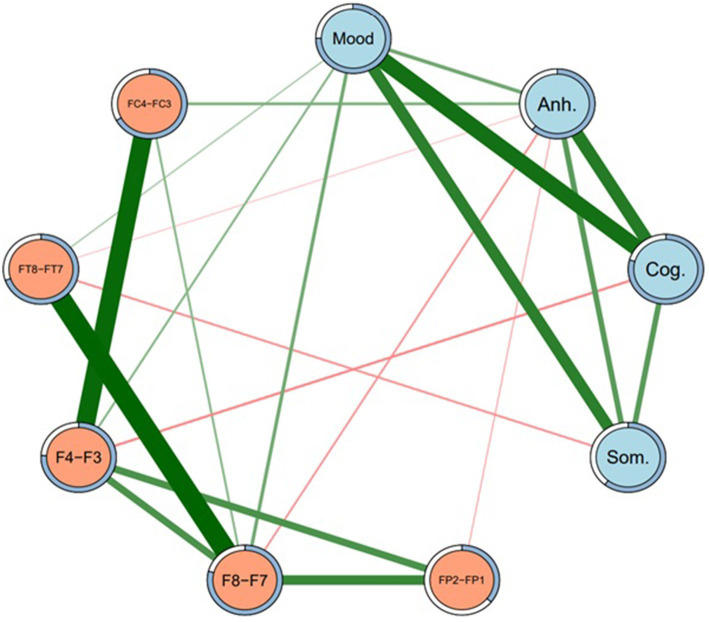
Network structure of depression subtypes and EEG sites (Model 1). Regularized network at gamma level 0.5. Mood = Depressed mood, Anh. = Anhedonia, Cog. = Cognitive, Som. = Somatic. Green lines represent positive edges. Thicker lines indicate stronger associations. Rings around each node represent predictability (i.e., proportion of variance in each node explained by nodes that it shares an edge with).

A regularized model at gamma level 0 (Model 1) identified a number of non-zero DB subtype-EEG edges. Non-zero edges included: between Depressed mood and F8-F7, F4-F3, and FT8-FT7 (positive associations); between Anhedonia and FC4-FC3 (positive association); between Anhedonia and FP2-FP1, F8-F7, and FT8-FT7 (all negative associations); between Cognitive subtype and F4-F3 (negative association); and between Somatic subtype and FT8-FT7 (negative association). The network structure of Model 1 is presented in Figure 1, and its edge weights and edge stability are reported in Table 3.

**Table 3:** Depression subtype-EEG edge weights and edge stability.

Depression subtype	EEG site				
	F4-F3	F8-F7	FC4-FC3	FP2-FP1	FT8-FT7
Depressed mood	Model 1				
	0.046 (66.7%)	0.088 (79.8%)	—	—	0.019 (67.2%)
	Model 3				
	0.012 (53.7%)	0.065 (84.5%)	0.006 (31.7%)	—	—
	Model 1				
Anhedonia	—	−0.035 (49.6%)	0.066 (82.3%)	−0.016 (58.4%)	−0.006 (48.3%)
	Model 3				
	—	—	0.017 (63.1%)	—	—
Cognitive^a^	Model 1				
	−0.051 (67.2%)	—	—	—	—
Somatic^a^	Model 1				
	—	—	—	—	−0.040 (60.6%)

Model 1 = regularized network with gamma 0. Model 3 = regularized network with gamma 0.5. Values are unstandardized edge weight sample coefficients. Proportion of 2,500 bootstrapped samples that the edge was replicated as non-zero (i.e., edge stability) in parentheses. In empty cells, no edge was detected.

a No edges detected in Model 3.

**Figure 2:**
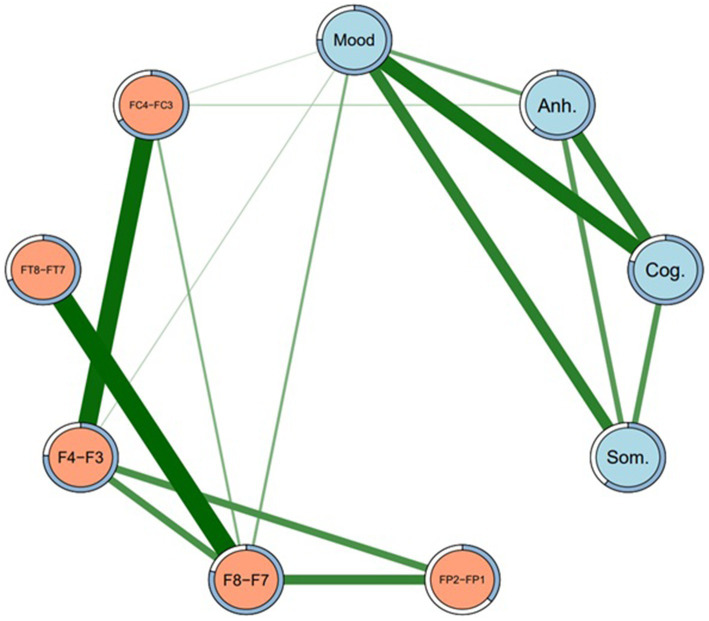
Network structure of depression subtypes and EEG sites (Model 3). Regularized network at gamma level 0.5. Mood = Depressed mood, Anh. = Anhedonia, Cog. = Cognitive, Som. = Somatic. Green lines represent positive edges. Thicker lines indicate stronger associations. Rings around each node represent predictability (i.e., proportion of variance in each node explained by nodes that it shares an edge with).

Because a large number of subtype-EEG edges in Model 1 were detected as non-zero, the regularized network was re-run at gamma 0.25 (Model 2) and again at gamma 0.5 (Model 3), such that these networks were increasingly more conservative and thus “truer” representations of the MDD subtype-EEG network, and acted as a sensitivity analysis to determine possible false positive edges (76). In Model 2, many non-zero DB subtype-EEG edges dropped to zero, except edges between depressed mood and F8-F7, F4-F3, and FC4-FC3, and Anhedonia-FC4-FC3. Similarly, in Model 3 these same four edges remained non-zero, with identical sample edge weights and similar stability across bootstrapped samples to Model 2. The association between depressed mood subtype and F8-F7 appeared the most stable DB-EEG association across networks. Some edges, however, were small in effect and not significantly greater than other edges in their respective networks. Models 2 and 3 were also identical in density (44.4% of edges were non-zero) and less dense than Model 1 (58.3% non-zero edges). As such, of these two more conservative models, only the network structure of the more conservative Model 3 is visually presented, in Figure 2. Its edge weights and edge stability values are presented in Table 3.

An unregularized model (Model 4) was also run. Some previous research has compared both unregularized and regularized networks (76) because unregularized models may reduce the detection of false positive edges (72). As expected, Model 4 was sparser (27.8% of edges were non-zero) than the regularized networks reported above. However, although the unregularized model also showed a non-zero positive edge between Depressed mood and F8-F7, this was less stable (it was replicated as non-zero in just 49.3% of 2,500 bootstrapped samples) than in the regularized models (see Table 3). Other DB subtype-EEG edges were reduced to zero in the unregularized model. Thus, although the unregularized model was more parsimonious, the depressed mood-F8-F7 edge was less stable in the unregularized model, and therefore less interpretable, than its regularized counterparts. The unregularized network structure is therefore not reported.

As well as presenting all of the edge weights with each EEG site for all four DB subtypes, Table 3 also allows for a comparison of the *direction* of those associations, i.e., whether they were direct (positive) or inverse (negative). Because the DB subtype scores were always positive, a direct network association (green lines in Figures 1, 2) indicates that an FLA datum was also positive (i.e., Rα > Lα), which indicates L > R overall activation. Similarly, an inverse network association indicates R > L overall activation, shown by red lines in Figures 1, 2.

As indicated in Table 3, depressed mood had only direct associations with EEG sites, but the other three DB subtypes had inverse associations with most EEG sites, apart from a single direct association between Anhedonia and FC4-FC3, which was present under Models 1 and 3. The strength of these associations may be understood by the figures in parentheses, which are indicative of the stability of each association on the basis of 2,500 bootstrapped samples. These directions of association are summarized in Table 4, and provide an insight into the ways that each of these four DB subtypes was congruent, or not, with the traditional FLA-depression hypothesis.

**Table 4:** DB subtype-EEG AA associations and the FLA-depression hypothesis.

DB subtype	FLA-depression congruent associations	FLA-depression non-congruent associations
Depressed mood		F4-F3*, F8-F7*, FT8-FT7, FC4-FC3
Anhedonia	F8-F7, FP2-FP1, FT8-FT7	FC4-FC3*
Cognitive depression	F4-F3	
Somatic depression	FT8-FT7	

* Association was supported across Models 1 and 2.

## Discussion

Three major findings emerged from this research. First, each of the four DB subtypes exhibited distinct neurophysiological profiles defined by FLA, not only in terms of the presence of associations between DB subtypes and the specific EEG-FLA sites examined, but also in terms of the direction (i.e., direct vs. inverse) of those associations. Second, as shown in Table 2, columns 4, 5, the sample’s scores for each of the four DB subtypes were significantly different to each other, implying that they represented different aspects of the overall MDD symptomatology presented in the SDS, and that they had varying associations with the SDS total score despite (as would be expected) all being significantly correlated with that total score (col. 6). Third, as mentioned in the Introduction, the previous FLA-MDD findings have not been consistent, and one possible explanation for that inconsistency may have been the mix of DB subtypes in the samples of participants. That is, Figure 1 shows both direct and inverse associations between DB subtypes and EEG site FLA data, but the prevalence of each of these DB subtypes in past samples is unknown, and could potentially confound the overall FLA-MDD associations reported in those studies. These findings have clinical implications, which will be discussed below.

Considering the first two findings (that each of the four DB subtypes exhibited distinct score and neurophysiological profiles defined by FLA), the associations between Depressed mood and F8-F7, and F4-F3 were the most stable across networks (i.e., Figures 1, 2), and were both in a positive direction. As explained above, these positive associations indicate greater overall (i.e., non-alpha) electrical activity in F7 and F3 (left hemisphere) than in F8 and F4 (right hemisphere), emphasizing the L > R activation nature of this DB subtype. Second, Depressed mood was also characterized by greater activation in FT7 rather than FT8, although that was not common across Models 1 and 3. By comparison, Anhedonia had a mixed set of L > R results (FC4-FC3 in Models 1 and 3) plus R > L results for F8-F7, FP2-FP1, and FT8-FT7, although only in Model 1. Cognitive depression and Somatic depression had single R > L results for F4-F3 and FT8-FT7, respectively. Thus, there were differences in the FLA signatures of each of these four DB subtypes, arguing that they may be described as differentially associating with the 10 EEG sites measured here. That is, as shown in Table 4, Depressed mood is associated with left hemisphere activation in four EEG sites, Anhedonia is associated with right hemisphere activation in three EEG sites and left hemisphere activation in one left EEG site, Cognitive and Somatic depression are associated with right hemisphere activation in a single EEG site each. These EEG findings complement those reported in Table 2, that showed the significant difference in mean scores for each of the four DB subtypes, by underscoring the different depression profile that each of these four DB’s represented, which included different symptomatology content (Table 1), as well as different response patterns (Table 2) and different neurophysiological characteristics (Tables 3, 4; Figures 1, 2).

The third major finding was in relation to the FLA-depression hypothesis, and these results offer a plausible argument for the inconsistency in the previous literature regarding this hypothesis by finding no significant correlations between any of the FLA EEG data and the SDS total score, but several meaningful associations between the four DB subtypes and EEG FLA data. This difference in results is also a reflection of a different data analysis approach and methodology. That is, the FLA-SDS total score finding reported here was similar to those obtained in previous research using traditional correlational procedures or ANOVA models based upon a depression total score or a dichotomous diagnosis, used almost universally in previous studies of the FLA-depression hypothesis. By contrast, the findings reported here for the four DB subtypes were based upon network analysis, which assumes a more interactive model of symptomatology (i.e., including all the DB subtypes and the EEG FLA data). As succinctly described by Borsboom and Cramer [(53), p. 96]: “Instead of interpreting symptoms as a function of a set of underlying/latent disorders, the network approach conceptualizes symptoms as mutually interacting, often reciprocally reinforcing, elements of a complex network.” Extending this logic to the present study, all of the EEG sites, their FLA metrics, and the four DB subtypes represent “symptoms” rather than one set of latent or underlying disorders (i.e., EEG activity, representing BIS/BAS processes) causing a set of symptoms (i.e., DB subtypes).

As mentioned in the Introduction, there is not universal support for the FLA-depression hypothesis, and one recent study exemplifies that challenge to the association between AA and MDD by reporting that, in a mixed-sex non-clinical sample of 99 young volunteers, FLA was not significantly associated with depression (80). A recent meta-analytic review of the FLA-depression hypothesis (81) found a small and nonsignificant effect size, and an even more recent multiverse analysis (i.e., 270 forms of data analysis of every combination of EEG sites) concluded that the results were “incompatible with the presence of moderate to strong relationship” between FLA and depression [(82), p. 19]. Of particular relevance to the results of the present study, that multiverse analysis found that only 13 of the 270 analyses produced significant results (which would have been expected by chance using *p* < 0.05 as a criterion), five of which supported the FLA-depression hypothesis, two failed to show any asymmetry effects, and six showed the opposite association (i.e., L > R instead of R > L electrical activity). Concomitantly, it has been suggested that earlier supportive reviews of the FLA-depression hypothesis may have been subject to publication bias (29). Finally, as suggested above, these inconclusive previous findings may have been influenced by different sample levels of the four DB subtypes examined here, or a different approach to the issue of explaining the association between EEG site activity and DB subtypes (i.e., network analysis).

The FLA-depression hypothesis rests, to some extent, on the distinctive nature and influence of the BIS vs. BAS upon behavior. If the BAS-BIS hypothesis was accepted for the purposes of discussion, then this would imply that depressed mood was directly associated with increased BAS activity in this study. Although that interpretation appears contradictory to the BAS-BIS-Depression hypothesis that argues the BAS (left hemisphere) is linked with approach activities, and the BIS (right hemisphere) is associated with the kind of behavioral withdrawal that underlies much depressive behavior (36, 37, 83), it is worth reflecting that Reznik and Allen [(51), p. 3] noted that the BIS may include some approach behaviors, and the BAS may be linked with safety-seeking, which could be considered as a withdrawal behavior. Bearing in mind that “the isomorphic mapping of BIS and BAS systems to withdrawal and approach systems” has been challenged, and that the correlations between BAS and left frontal activity “are of an insufficient magnitude” to draw any conclusions on this hemispheric dominance-depression association [(51), p. 3], reliance on the BIS-BAS-Depression hypothesis to explain these findings may be unwise if that reliance was based upon supposed hemispheric location of these two behavior systems.

In summary, using network analysis, the results from Models 1 and 3, and portrayed in Figures 1, 2, provide a defensible neurophysiological structure of the ways that the four DB subtypes examined here are differentially related to electrical activity in specific brain sites. As such, these results provide support for the original argument that, as well as being distinct from each other in terms of their symptom content (11), these four DB subtypes also have different neurophysiological characteristics. It is also of interest to note the associations between the four DB subtypes shown in both Figures 1, 2, suggesting that they reflect an underlying connection that may contribute to global MDD.

Limitations of this study include the sample size and characteristics, the cross-sectional nature of the study, and the reliance on self-report data for depression. Addressing these factors would enhance generalizability of data from future studies, and a larger sample of clinically significantly depressed participants would allow for network analysis of that subgroup. Although that was not possible here, the inclusion of all (depressed, not depressed) participants provided a basis for considering the MDD subtype network analysis within a community sample. Although there are limitations upon the application of network analysis, the results reported here revealed FLA-DB subtype associations that correlation analyses could not, suggesting that network analysis may be a worthwhile avenue for future investigations of the FLA-MDD hypothesis. The collection of comorbidity data regarding anxiety or other disorders, and medication status, would also enhance future research. Although this study was purposely focused on the frontal lobes, principally because of the large amount of previous research into the FLA hypothesis, future research could also consider the effects of asymmetry on depression across other brain regions, such as the temporal lobes, where previous research has found links between asymmetry and depression (84, 85).

In terms of clinical relevance, these findings add to the existing literature regarding the heterogeneity of MDD by suggesting that different forms of depressive behavior may be associated with actual neurological differences, and therefore represent potentially more valid targets of diagnosis than global MDD alone. As well as the different neurophysiological profiles for these four DB subtypes that are reported here, the symptomatologic differences (Table 1) between them argue for not only differential assessment processes, but also more subtype-focused treatment planning than is often applied to global MDD diagnoses. It has been demonstrated for some time that first-line antidepressant, psychotherapeutic, and lifestyle treatments for global MDD are limited in their efficacy (86–88), and recent reviews have not challenged that evaluation (89–91). What all these reviews had in common is a recommendation to develop the question of “What works best for whom?” approach by individualizing diagnosis and consequent treatment (92). The definition of the four DB subtypes examined here, plus the demonstration of their neurophysiological profiles, sits alongside their symptom content, and statistical distinction in occurrence, to provide a step towards finding at least part of the answer to that question.

In conclusion, two steps were taken in this research to investigate and clarify the FLA-depression hypothesis. First, based upon repeated calls in the literature for developing a model of depression that reflects the heterogeneity of MDD, DB subtypes were used as the metric of depression, rather than total scores on a scale or a dichotomous diagnosis, thus enabling a more detailed consideration of the nature of the FLA-depression relationship. Second, because of the conceptual limitations of the traditional view of psychopathology that conceives disorder as causing symptomatology, a more reciprocal model was adopted, that included EEG site activity, resultant FLA data, and DB subtypes as potentially associated in more intimate ways (the “mutually interacting, often reciprocally reinforcing, elements of a complex network” described by 54). On that basis, the concept of depression arising from dominance of the BIS over the BAS, and the resultant asymmetry in frontal lobe hemispheric activity, might be replaced by an interactive model of depression wherein all these aspects were influencing each other, producing a depressive-like state that represents part of the natural homeostatic processes underlying behavior, even if those processes have become exaggerated to the point of disorder, perhaps by the actions of chronic stressors (93).

## Data availability statement

The raw data supporting the conclusions of this article will be made available by the authors, without undue reservation.

## Ethics statement

The studies involving human participants were reviewed and approved by University of New Engaland Human Research Ethics Committee. The patients/participants provided their written informed consent to participate in this study.

## Author contributions

CS and VB designed the study, analyzed the data, and drafted the manuscript. EJ collected the data. WA performed the network analysis. SS analyzed the EEG data and drafted the manuscript. LA oversaw the project. All authors contributed to the article and approved the submitted version.

## Conflict of interest

The authors declare that the research was conducted in the absence of any commercial or financial relationships that could be construed as a potential conflict of interest.

## Publisher’s note

All claims expressed in this article are solely those of the authors and do not necessarily represent those of their affiliated organizations, or those of the publisher, the editors and the reviewers. Any product that may be evaluated in this article, or claim that may be made by its manufacturer, is not guaranteed or endorsed by the publisher.
